# Contrasting Effects of Probiotic Yogurt and Brown Yogurt on the Urinary Metabolites and Gut Microbial Function of Healthy Adults

**DOI:** 10.1002/fsn3.71144

**Published:** 2025-11-03

**Authors:** Si Ting Chen, Tie Min Jiang, Yu Chun Wang, Xia Qi Xiong, Jun Ying Zhao, Li Jun Chen

**Affiliations:** ^1^ College of Chemistry and Bioengineering Guilin University of Technology Guilin Guangxi P. R. China; ^2^ National Engineering Center of Dairy for Maternal and Child Health Beijing Sanyuan Foods Co. Ltd. Beijing P. R. China; ^3^ Liuzhou Sanyuan Tianai Dairy Co., Ltd. Liuzhou Guangxi P. R. China

**Keywords:** gut microbiota, healthy adult, urinary metabolome, yogurt

## Abstract

Consuming yogurt is beneficial for healthy people, but few studies have evaluated the interactions among yogurt intake, gut microbiota, and the metabolism of healthy hosts. We aimed to compare the effects of probiotic yogurt (Yi Jun Duo; YJD) and brown yogurt (Shao Suan Nai; SSN) on urinary metabolite concentrations and gut microbial composition and function in healthy adults. The SSN was produced by *Streptococcus thermophilus* S4.02 and low‐fat (1.2%) milk, which was hydrolyzed using galactosidase and browned, and the YJD was whole milk fermented by a mixture of *Lactobacillus bulgaricus* , *S. thermophilus* , *L. acidophilus* , and *Bifidobacterium lactis* . Forty‐six healthy adults (*n* = 23/group) consumed the yogurts for 28 days. NMR metabolomics was then used to study the differences in the concentrations of urinary metabolites, and the composition of the intestinal microbiota was characterized using 16S rRNA amplicon sequencing. SSN consumption significantly increased the urinary concentrations of methylamine, O‐phosphocholine, trimethylamine N‐oxide (TMAO), and 3‐hydroxyisobutyrate, and reduced the abundances of the Pasteurellaceae, Enterobacteriaceae, *Dorea*, *Megamonas*, *Haemophilus*, and *Shuttleworthia*. YJD consumption reduced the concentrations of O‐phosphocholine, fumarate, and tryptophan, reduced the abundance of *Collinsella*, and increased the urea concentration and the abundances of the Porphyromonadaceae and *Parabacteroides*. Twelve metabolites differed significantly in concentration between the two groups. SSN and YJD also had differing effects on carbohydrate metabolism (pyruvate metabolism, TCA cycle, and glycolysis/gluconeogenesis) and the amino acid metabolism pathway (phenylalanine, tyrosine, and tryptophan biosynthesis, and metabolism of histidine and tyrosine), principally by regulating the TMAO and amino acid metabolism of the intestinal bacteria.

## Graphical abstract

We identified differing metabolic responses to different types of yogurt. Brown yogurt mainly modulated pathways associated with human and gut microbial ammonia metabolism, whereas probiotic yogurt mainly affected amino acid metabolism.

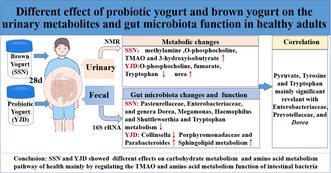

## 1 Introduction

Yogurt consumption is associated with favorable health outcomes, including lower risks of cancer (Yang et al. 2019) and type 2 diabetes (Daniel et al. 2022), a better metabolic profile (Wang et al. 2013), weight maintenance, and superior cardiovascular (Buziau et al. 2019) and bone health (Ong et al. 2020). Preliminary studies have suggested that alterations to the structure and function of the gut microbiota might be at least in part responsible for these beneficial associations (Miller et al. 2021; Perazza et al. 2020; He et al. 2022; Yılmaz et al. 2019), and there is an increasing amount of evidence that the gut microbiota contributes to host health status (Marchesi et al. 2016; Cani 2018). Gut microbial composition and function are typically stable, but may be affected by several factors, including age and diet, and in turn affect host metabolism via the gut–organ axis (David et al. 2014; Cani 2014; Ahlawat et al. 2020).

Yogurt is a fermented food and a vector for a variety of probiotics, and many studies have shown that it can influence the gut microbial composition of healthy people (Lisko et al. 2017; Suzuki et al. 2017; Burton et al. 2017; Elise et al. 2007), as well as that of individuals with a variety of conditions, including diabetes (Miller et al. 2021), inflammatory bowel disease (Yılmaz et al. 2019), and overweight (Rahayu et al. 2021). However, few studies have assessed the interplay between yogurt intake, gut microbial composition, and host metabolism in healthy humans (Lee et al. 2021). Of note, the beneficial effects of yogurt may vary according to the type of yogurt, and depend on the species and strains of lactic acid‐generating bacteria used for fermentation (Redondo‐Useros et al. 2019; Jiang et al. 2022), as well as the animal species from which the milk originates (Rettedal et al. 2019; Redondo et al. 2019; Aljutaily et al. 2020).

The principal objective of the present study was to evaluate the influence of two types of yogurt, a probiotic yogurt (Yi Jun Duo; YJD) and brown yogurt (Shao Suan Nai; SSN), on the metabolism in healthy adults, and to identify potential underlying mechanisms. Brown yogurt, which originated in the Caucasus region of Russia in the mid‐16th century, is a fermented dairy product that is brown and has an appealing flavor. It has become popular in the Chinese market in a variety of different forms since 2017 (Yu et al. 2020). YJD and SSN have different microbial, fatty acid, carbohydrate, and fermented metabolite compositions (Jiang et al. 2022). Therefore, we speculated that differing metabolic responses would be associated with the consumption of these two products.

## 2 Materials and Methods

### 2.1 Participants and Samples

Forty‐six healthy adults (*n* = 23/group, with 8 men in each of the SSN and YJD groups) were selected to participate in a randomized, open‐label study of the effects of SSN and YJD yogurts on the gut microbiota (Jiang et al. 2022). The study was approved by the Ethics Committee of the Staff Hospital at Luoyang North Enterprise Group Co. Ltd. (approval number #2018032). All participants were enrolled in Beijing and written informed consent was obtained from each. None had a known metabolic disease or gastrointestinal disorder, and none had taken antibiotics during the 3 months preceding the study.

From day 0, all the participants consumed one 100‐mL serving of the assigned yogurt in both the morning and evening over a 28‐day period, during which they were allowed to maintain their regular lifestyle and diet, but were asked to abstain from consuming other products containing probiotics or prebiotics and taking antibiotics. Each of the participants was asked to provide a fecal sample on days 0 (baseline or d0) and 28 (d28) and a urine sample every week, and to complete a diet diary covering the 72‐h period prior to the day of sample collection. The principal characteristics of the participants are presented in Table 1. Ninety urine and 90 fecal samples were selected for analysis.

**TABLE 1:** Characteristics of the volunteers and their daily dietary intake.

Variable	SSN	YJD	*p*
Total (*n*)	23	23	
Gender (*n*)			
Male	8	8	ns
Female	15	15	
Age (mean ± SD years)	35.4 ± 7.5	36.4 ± 8.0	ns
BMI (mean ± SD kg/m^2^)	24.2 ± 3.5	23.2 ± 2.8	ns
Dietary intake (mean ± SD g/day)			
Grain staples	564.7 ± 472.4	589.7 ± 318.9	ns
Vegetables	361.5 ± 203.1	384.1 ± 247.4	ns
Fruits	106.4 ± 108.8	123.0 ± 131.0	ns
Meat	180 ± 184.5	279.1 ± 373.4	ns
Seafood	49.7 ± 99.9	29.6 ± 162.3	**
Eggs	43.9 ± 32.8	40.5 ± 39.3	ns
Beans and nuts	45.9 ± 51.3	78.9 ± 94.5	ns
Oil	19.7 ± 20.4	15.9 ± 16.9	ns

*Note:* Data are presented as mean ± standard deviation (SD) or *n*. Statistical analysis was performed using the Mann–Whitney *U* test. SSN: brown yogurt, Shao Suan Nai; YJD: probiotic yogurt, Yi Jun Duo.

Abbreviation: ns, not significant.

** *p* < 0.01.

The experimental yogurt products (SSN and YJD) were provided by Beijing Sanyuan Foods Co. Ltd. (Beijing, China). The SSN was created by *Streptococcus thermophilus* S4.02 from low‐fat (1.2%) milk, which was hydrolyzed using galactosidase and browned, and the YJD was obtained by the fermentation of whole milk by *Lactobacillus bulgaricus* , *S. thermophilus* , *L. acidophilus* , and *Bifidobacterium lactis* (3.5% fat). The protein content of both yogurts was 3.0%, and the carbohydrate contents were 12.0% and 10.8% for the SSN and YJD, respectively. The numbers of lactic acid‐generating bacteria present were 5.0 × 10^9^ cfu/g for SSN and 3.2 × 10^9^ cfu/g for YJD.

### 2.2 Analysis of Urinary Metabolites

Urinary metabolites were analyzed using proton–nuclear magnetic resonance (NMR). The samples were treated, and data were acquired and processed using previously described methods (Jiang et al. 2022). First, freeze‐dried samples were suspended in 1 mL of purified water and filtered using 3‐kDa ultrafilters (Merck Millipore, USA). Second, 450 μL of each filtrate was transferred to a clean, 2‐mL centrifuge tube, and then 50 μL of 4,4‐dimethyl‐4‐silapentane‐1‐sulfonic acid standard solution (Anachro, Canada) was added. Finally, the mixed samples were transferred to 5‐mm NMR tubes (Norell, USA) and analyzed using an AV III 600 MHz spectrometer (Bruker GmbH, Rheinstetten, Germany). The first increment of a 2D ^1^H–^1^H‐Nuclear Overhauser Effect Spectroscopy (NOESY) pulse sequence was used for the acquisition of ^1^H‐NMR data and the suppression of the solvent signal. We used a 100‐ms mixing time and a 990‐ms pre‐saturation period (~80 Hz gammaB1), a temperature of 25°C, and 128 scans over a period of 15 min.

The raw data were pre‐processed using the processing module in Chenomx NMR Suite 8.3. (Chenomx Inc., Edmonton, Canada). All the ^1^H‐NMR spectra were compared with the internal standard and 4,4‐dimethyl‐4‐silapentane‐1‐sulfonic acid and analyzed with reference to the Chenomx Compound Library. From 180 spectra generated, 65 metabolites were identified and quantified (in mmol/L). The concentrations of all the metabolites were normalized by weight across all the parallel samples before being used in multivariate analyses.

To characterize the differences in the metabolite composition of the two groups, multivariate analysis was performed using partial least squares discriminant analysis (PLS‐DA). Kyoto Encyclopedia of Genes and Genomes (KEGG) pathway analysis was then used to identify the pathways that were significantly differentially affected by yogurt type in the MetaboAnalyst web tool (www.metaboanalyst.ca).

### 2.3 Metabolic Functions of the Gut Microbiota

The gut microbial compositions of the 90 fecal samples were analyzed using next‐generation sequencing. In brief, genomic DNA was extracted from 100‐mg aliquots of homogenized fecal samples, and the V3–V4 region of the 16S rRNA genes was amplified by PCR. Subsequently, NEB Next Ultra DNA Library Prep Kit for Illumina (NEB, USA) was used to generate DNA libraries for sequencing according to the manufacturer's instructions. Lastly, the taxonomic assignment of representative amplicon sequence variants (ASVs) obtained from 2‐ × 250‐bp paired‐end sequencing data was performed using the Greengenes database v.13.8 (https://ftp.microbio.me/greengenes_release/gg_13_8_otus/taxonomy/) and the Ribosomal Database Project classifier.

Functional profiling of the 16S rRNA amplicons was performed using PICRUSt2 (v2.5.0) in Wekemo Bioincloud (https://www.bioincloud.tech). The ASV counts and representative sequences were uploaded as inputs. The parameters were set as follows: minimum sequence similarity threshold 0.8, minimum sample presence 1, hidden‐state prediction method ‘mp’, MinPath inference, and NSTI calculation enabled. The metabolic effects of the microbiota were predicted using PICRUSt2, and principal coordinates analysis (PCoA) was performed to predict the functional variation in KEGG orthology (KO) after the consumption of the two types of yogurt. The KOs were subjected to KEGG enrichment analyses using OmicShare tools (https://www.omicshare.com/tools), Kruskal–Wallis test for further analysis.

### 2.4 Statistical Analysis

For the metabolomics data, means and standard deviations were calculated, and paired *t*‐tests and one‐way analysis of covariance (ANCOVA) were used to identify the metabolites present at significantly different concentrations between the two groups in SPSS v.25.0 (IBM Inc., Armonk, NY, USA). *p* < 0.05 was regarded as indicating statistical significance. For gut microbial function, DESeq2 was used to identify significantly different features, using *p* < 0.05 and a fold‐change threshold of 150%. The relationships between metabolite concentrations and the gut microbial composition were evaluated using Spearman's rank correlation analysis, and an FDR‐adjusted *p*‐value (*q*‐value) < 0.1 was considered to be statistically significant.

## 3 Results

### 3.1 Metabolite Profiles Associated With Yogurt Type

**FIGURE 1:**
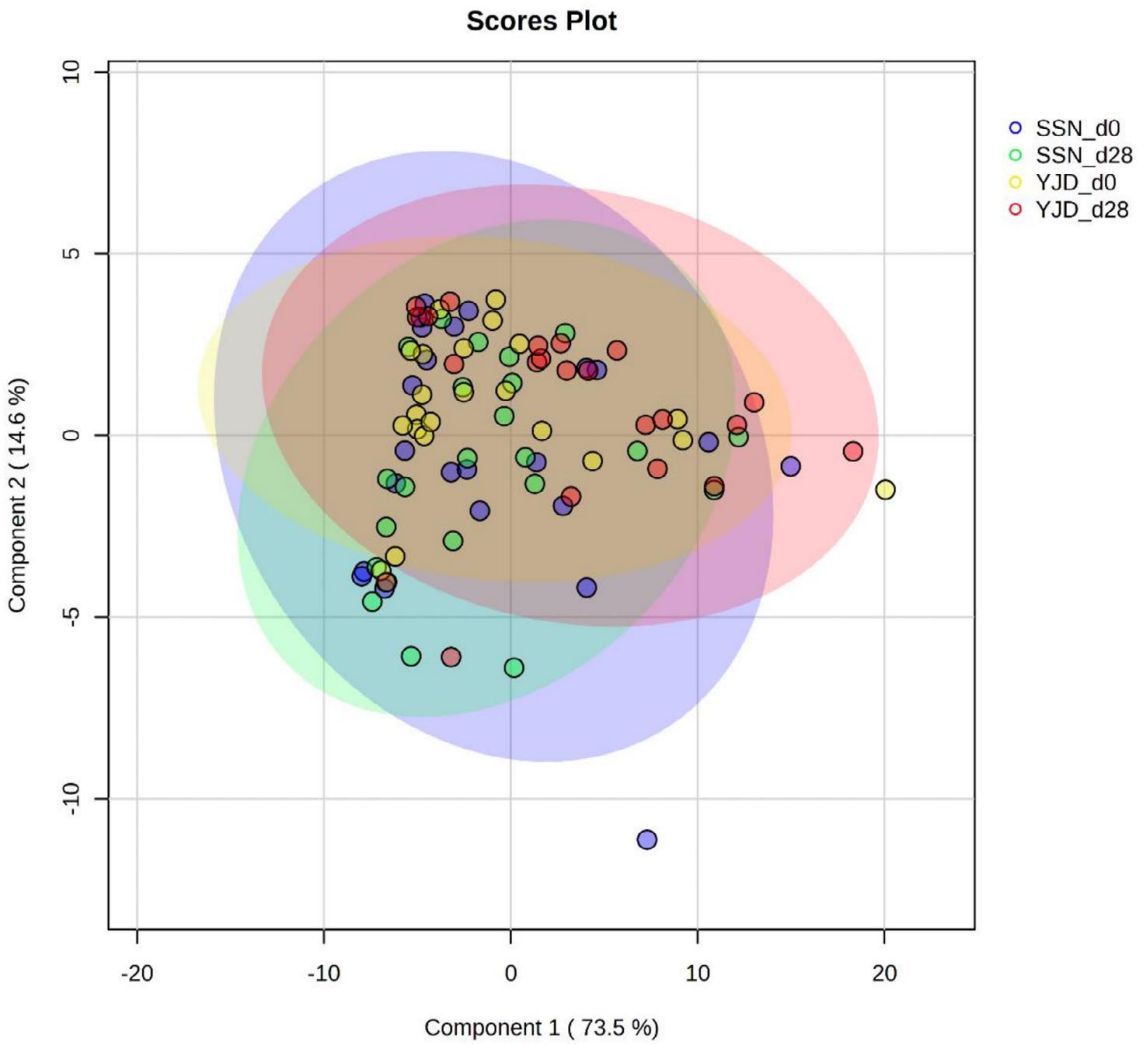
Partial least squares discriminant analysis score plot for the urine NMR spectra after the yogurt intervention. The data were Pareto‐scaled. The colored dots represent the specific urine samples and the rings represent 95% confidence regions. The groups are indicated using red dots (YJD_d28), green dots (SSN_d28), yellow dots (YJD_d0), and blue dots (SSN_d0).

**FIGURE 2:**
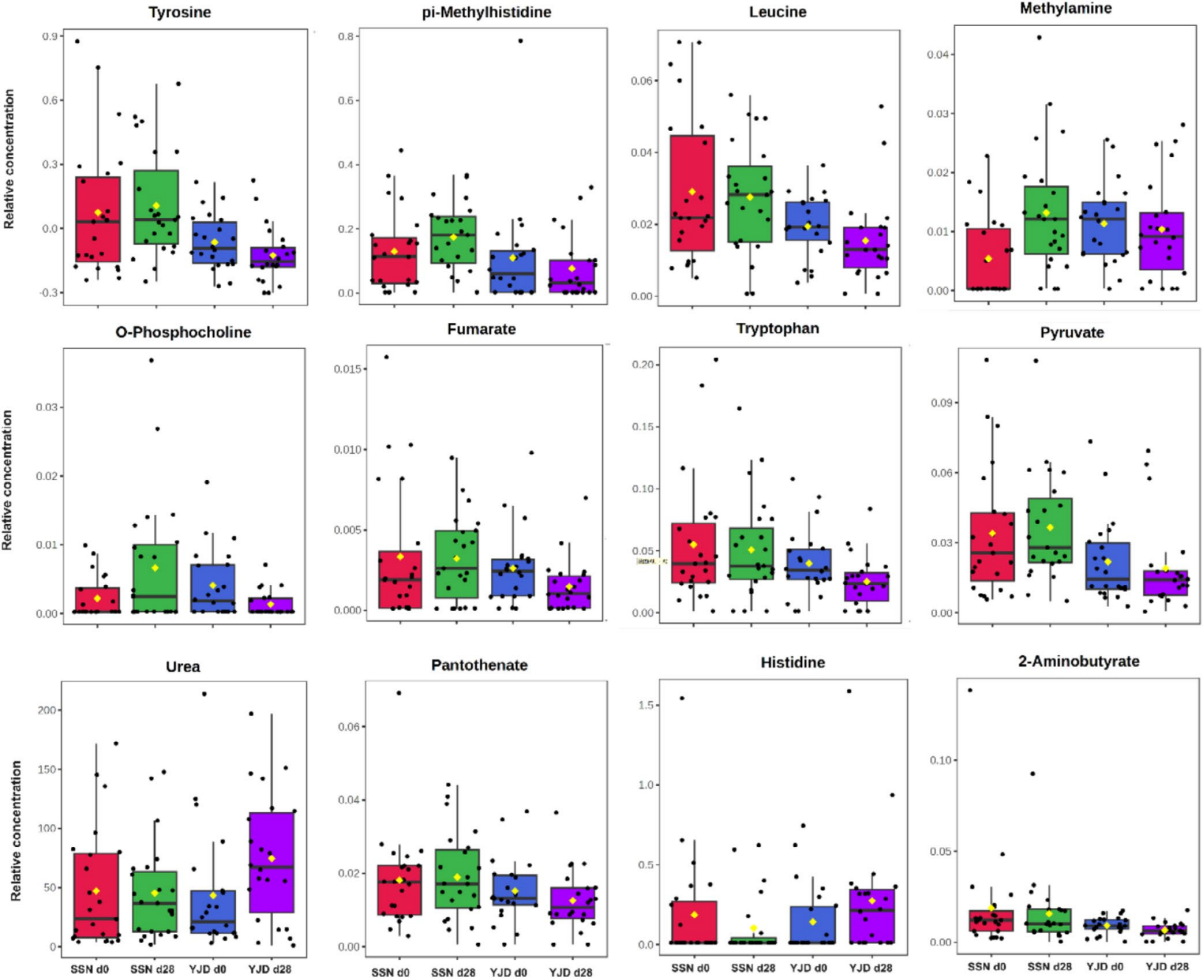
Box plots for the metabolites present at significantly different concentrations in the SSN and YJD groups after 28 days. The metabolites that were present at significantly different concentrations, according to paired *t*‐testing and one‐way analysis of covariance, were selected and the relative concentrations of each of these were plotted for the four subgroups.

**FIGURE 3:**
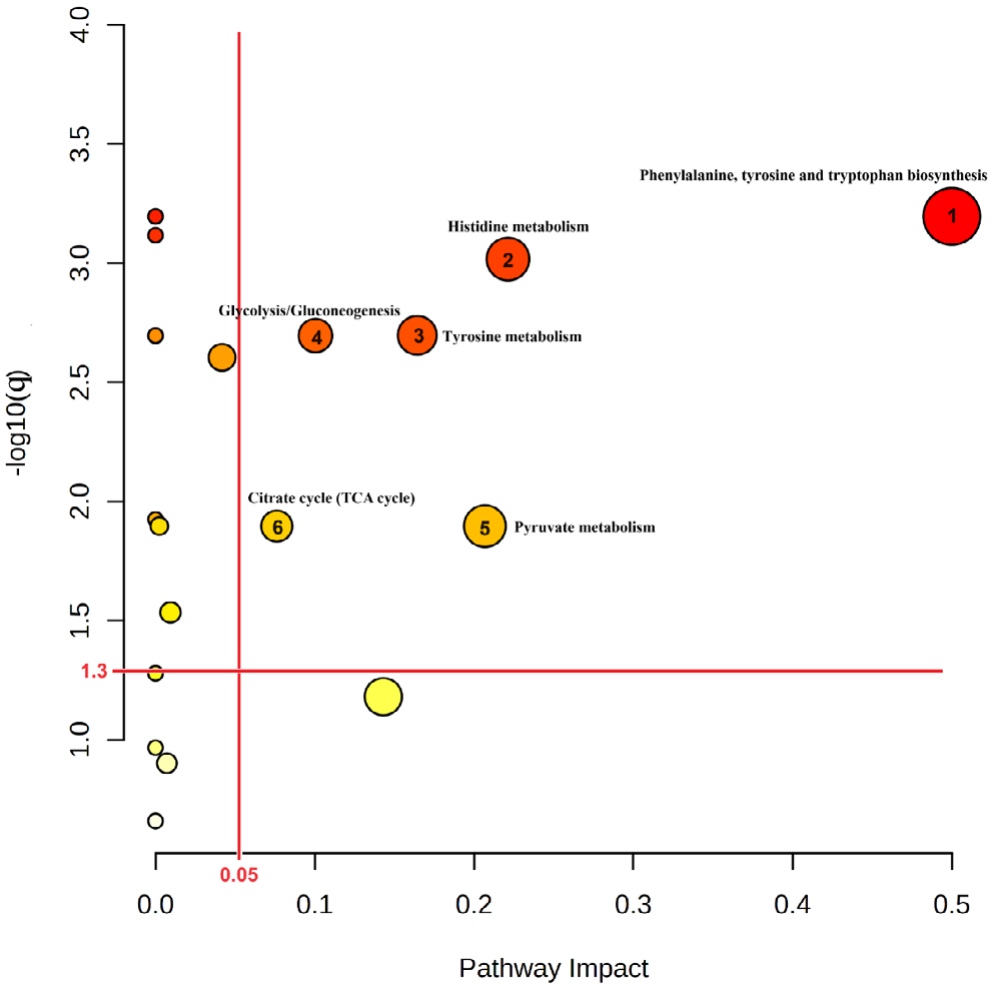
Results of the metabolic pathway analysis of the 12 metabolites present at different concentrations following the yogurt intervention: KEGG pathway analysis of the metabolites that were present at significantly different concentrations in the SSN and YJD groups after 28 days was performed using MetaboAnalyst. The *x*‐axis represents the pathway impact values from the pathway topology analysis and the *y*‐axis represents the −log10 (*q*‐value) generated from the pathway enrichment analysis. The node size reflects the pathway impact score and the node color indicates the *q*‐value (the redder the color is, the higher the level of significance was). Pathways with low *q*‐values and large pathway impact scores are considered to be highly influential. The red lines denote the thresholds on both axes for the identification of the most significant matched pathways for SSN‐d28 vs. YJD‐d28.

Sixty‐five known metabolites were identified in 90 urine samples from healthy adults using NMR analysis (Table S1). PLS‐DA analysis was used to assess whether specific metabolic signatures could be assigned to the two yogurt groups. As shown in Figure 1, the global metabolic profiles for the two groups differed in the two principal components PC1 and PC2, which accounted for 73.5% and 14.6% of the metabolites, respectively. SSN consumption was associated with significant increases in the concentrations of methylamine (MA), o‐phosphocholine (OPC), 3‐hydroxyisobutyrate (3‐HIB), and trimethylamine N‐oxide (TMAO); whereas YJD consumption was associated with significant decreases in the concentrations of tryptophan (Trp), OPC, fumarate, and a substantial increase in the concentration of urea (all *p* < 0.05, Table S1). ANCOVA adjusted for the baseline concentration of each compound showed that 12 metabolites were present at significantly different concentrations after SSN or YJD consumption (all *p* < 0.05). These metabolites are shown in Figure 2, in the order of *p*‐value, from low to high, and were tyrosine (Tyr), pi‐methylhistidine (pi‐MH), leucine (Leu), OPC, MA, fumarate, Trp, pyruvate, urea, pantothenate, histidine (His), and 2‐aminobutyrate (2‐AB). The major metabolic pathways that were linked to yogurt type were Phenylalanine, tyrosine, and tryptophan biosynthesis (ko00400); Tyrosine metabolism (ko00350); Pyruvate metabolism (ko00620); Histidine metabolism (ko00340); and Glycolysis (ko00010) (*q* < 0.05; Figure 3).

### 3.2 Changes in the Gut Microbial Composition and Function Associated With Yogurt Consumption

**FIGURE 4:**
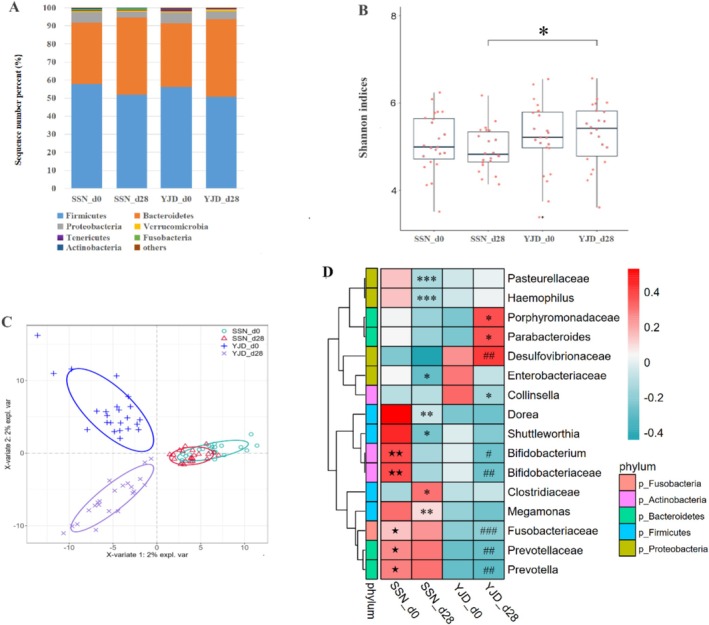
Changes in the intestinal microbial composition following yogurt consumption. (A) Relative abundances of the bacterial phyla in each group. (B) Boxplot of the Shannon indices. * Indicates a significant difference (Kruskal–Wallis test) after FDR. (C) Partial least‐squares discriminant analysis (PLS‐DA) score plot at operational taxonomic unit (ASV) level. The different symbols represent different fecal samples and the rings represent 95% confidence intervals. The groups are indicated as crosses (YJD_d28), triangles (SSN_d28), plus signs (YJD_d0), or circles (SSN_d0). (D) Heatmap of the bacterial taxa that showed significant differences in abundance, identified using Dunn's test with the Bonferroni adjustment (*p* < 0.05). * Indicates significant differences between d0 and d28 in the SSN or YJD group. # Indicates significant differences between d28 in the SSN or YJD groups. ★ Indicates a significant difference between SSN_d0 and YJD_d0.

A total of 4654 ASVs were identified in the 90 fecal samples and assigned to known bacterial taxonomy. The Firmicutes, Bacteroidetes, and Proteobacteria were the most common bacterial phyla (Figure 4A). The observed species, Faith PD, and Chao1 indices for the intestinal bacteria in the two groups at d28 had decreased slightly from baseline; but the Shannon indices of the YJD group increased during the intervention and were significantly higher than those of the SSN group (Figure 4B). Supervised PLS‐DA provided a summary of the gut microbial populations of the groups at the ASV level. The YJD group at d28 and d0 and the SSN groups were clearly separated, but there was no marked difference between the SSN group at d0 and d28 (Figure 4C). Further analysis by Dunn's test was performed to assess the effects of the two yogurt products on the intestinal microbiota of the participants.

The abundances of the Porphyromonadaceae family and its *Parabacteroides* genus were increased and that of *Collinsella* was reduced by YJD (*p* < 0.05), whereas the abundances of the families Pasteurellaceae and Enterobacteriaceae and the genera *Dorea*, *Megamonas*, *Haemophilus*, and *Shuttleworthia* were significantly reduced by SSN (*p* < 0.05). In addition, the abundances of Prevotellaceae, Bifidobacteriaceae, their members *Prevotella* and *Bifidobacterium*, and Fusobacteriaceae were significantly higher in the SSN group than in the YJD group after 28 days of consumption (*p* < 0.05; Figure 4D).

**FIGURE 5:**
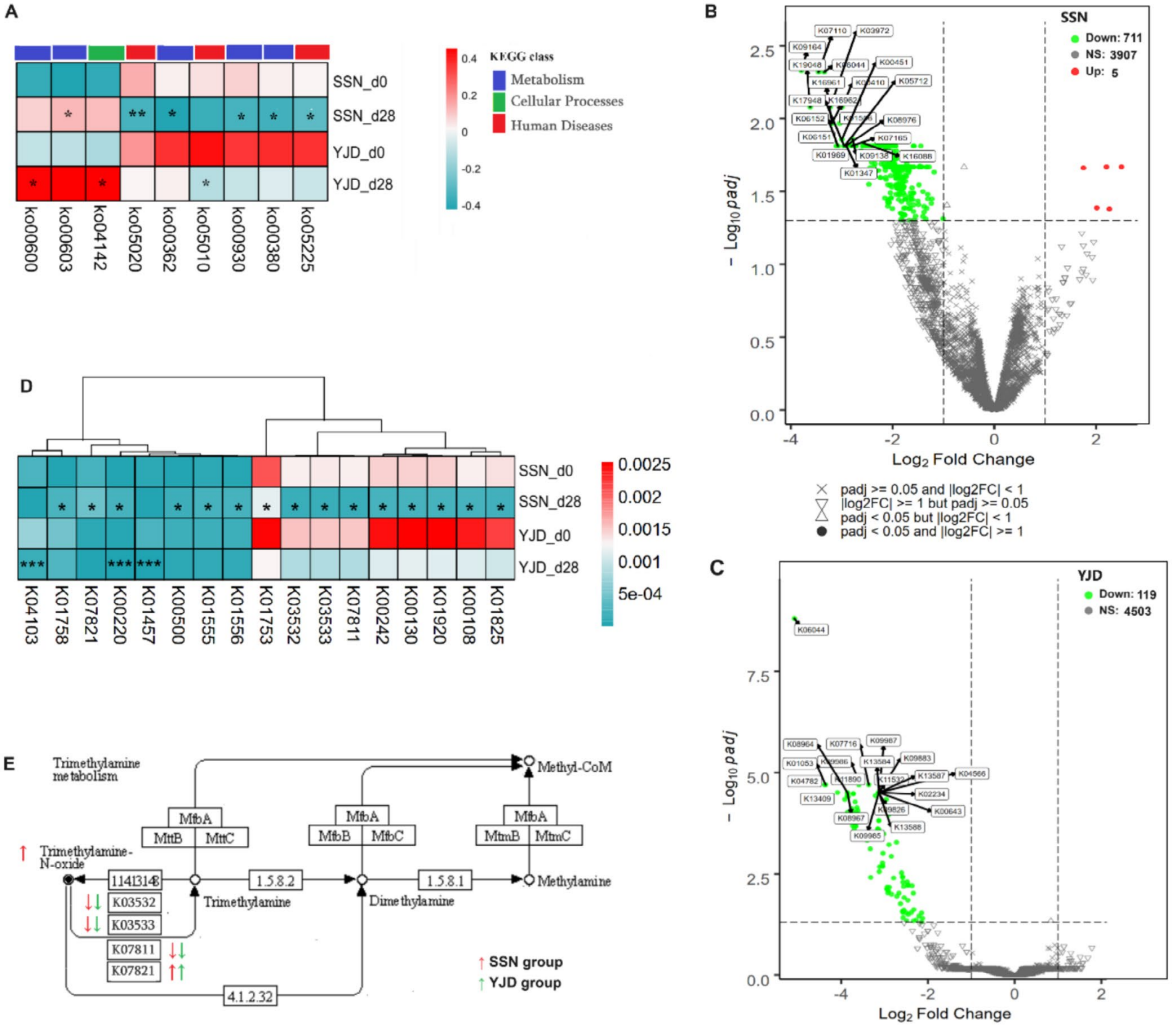
Effects of the yogurt intervention on intestinal microbiota function, predicted using PICRUSt2. (A) Heatmap of the significantly different KEGG pathways after SSN or YJD consumption, according to the Kruskal–Wallis test. (B, C) The DESeq2‐volcano plot of KEGG orthologies (KOs) for the SSN and YJD groups, which was based on fold change (FC) ≥ 2 or < 0.5 and an adjusted *p* < 0.05. (D) Heatmap of the significant enriched KOs derived from the metabolites that were present at different concentrations after yogurt consumption, according to Deseq2 analysis. The colors on the heatmap indicate the normalized median abundance (the redder the color is, the higher the abundance was; and the greener the color is, the lower the abundance was). (E) Trimethylamine metabolism. The red arrows represent the SSN group and the green arrows represent the YJD group; upward arrows indicate upregulation and downward arrows indicate downregulation. *p < 0.05, **p < 0.01, and ***p < 0.001 after FDR.

Bacterial metabolites influence host metabolism in numerous ways. The effects of the differential exposure to the metabolites were predicted using PICRUSt2. A number of metabolic pathways were found to be significantly affected using the Kruskal–Wallis test. Benzoate degradation (Ko00362), Tryptophan metabolism (Ko00380), Glycosphingolipid biosynthesis—globo and isoglobo series (Ko00603), Caprolactam degradation (Ko00930), Prion diseases (Ko05020), and Hepatocellular carcinoma (Ko05225) were affected by SSN consumption; whereas Sphingolipid metabolism (Ko00600), Lysosome (Ko04142), and Alzheimer's disease (Ko05010) were affected by YJD consumption (Figure 5A). The KOs were then analyzed to explore the relationships of the associated genes with urine metabolites. A total of 6442 KOs were identified and analyzed using a DESeq2‐volcano plot. Of these, 716 and 119 KOs were identified for the SSN and YJD groups, respectively (Figure 5B,C), and these were associated with significant enrichment of Tyrosine metabolism, Arginine and proline metabolism, and Phenylalanine metabolism (all *q* < 0.05; Table S2).

Of the identified differentially enriched KOs in KEGG, 17 were directly related to the metabolites present at different concentrations, as shown in Figure 2 and Table S1. K01555 (fahA; fumarylacetoacetase) and K00242 (sdhD/frdD; succinate dehydrogenase/fumarate reductase) were associated with fumarate metabolism, and both were significantly affected by SSN consumption (*q* < 0.05). K00220 (tyrC; cyclohexadienyl/prephenate dehydrogenase) was associated with Tyr metabolism, and there were opposite changes in the SSN and YJD groups (*q* < 0.05), whereas K00500 (PAH; phenylalanine‐4‐hydroxylase), which hydroxylates phenylalanine to Tyr, was upregulated by SSN consumption for 28 days (*q* < 0.05). K07811 (torA; TMAO reductase), K03532 (torC; TMAO reductase, cytochrome c‐type subunit torC), K03533 (torD; torA specific chaperone), and K07821 (torY; TMAO reductase, cytochrome c‐type subunit torY) were associated with the reduction of TMAO to trimethylamine (TMA), and the first three were significantly downregulated by SSN consumption (*q* < 0.05, Figure 5D,E). Pyruvate levels might be related to variations in K01753 (dsdA; D‐serine dehydratase) and K01758 (CTH; cystathionine gamma‐lyase) expression. K00453, a tryptophan 2,3‐dioxygenase (TDO2) responsible for catalyzing the conversion of Trp to N‐formylkynurenine, was downregulated by the consumption of SSN. Furthermore, K04103 (ipdC, indolepyruvate decarboxylase), K01556 (kynU, kynureninase), K01426 (amiE, amidase), and K01457 (atzF, allophanate hydrolase) may also be related to the Trp and urea levels (Figure 5D, Table S2).

**FIGURE 6:**
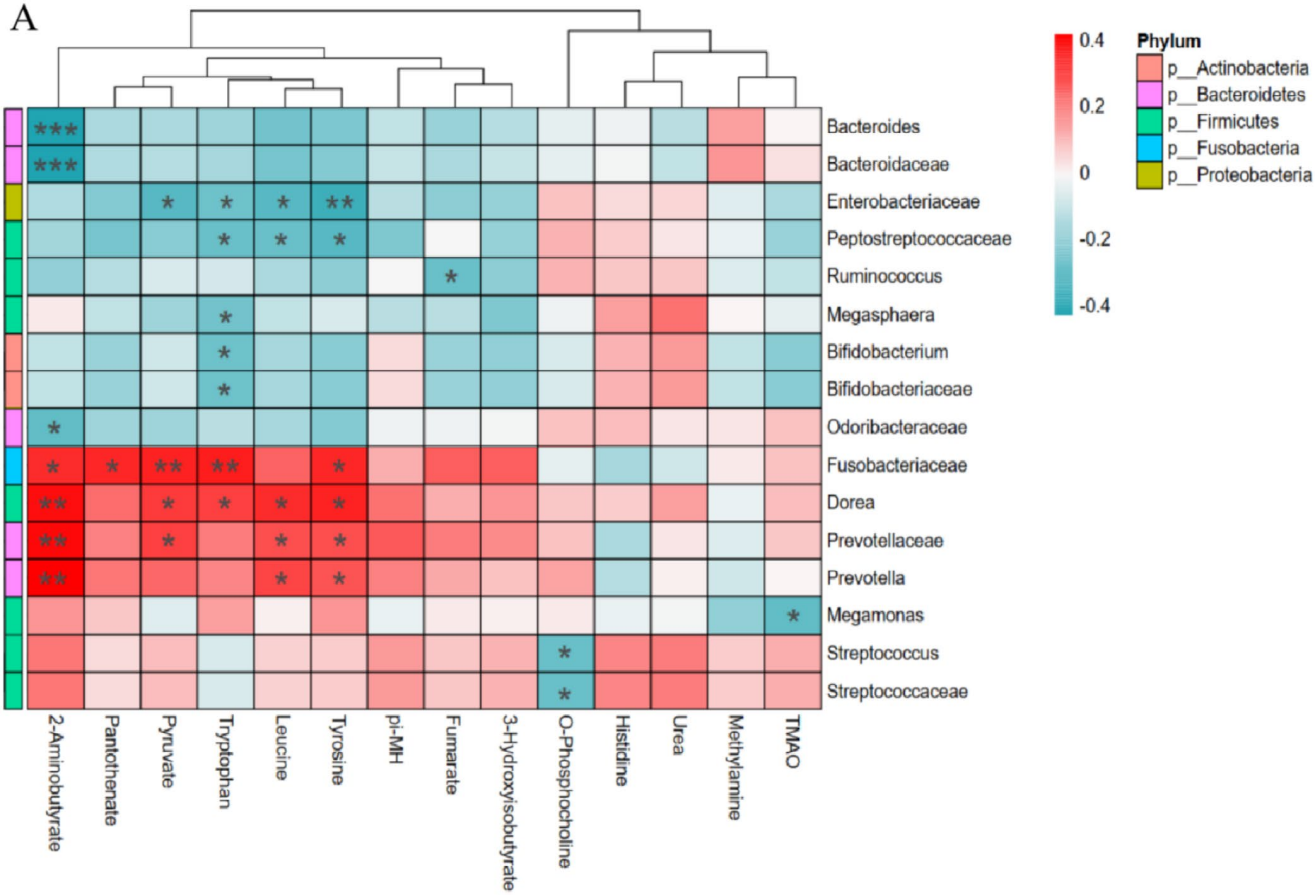
Relationships between gut bacterial taxa and urinary metabolites. (A) Heatmap of correlations between the abundances of gut microbial genera and the concentrations of specific urinary metabolites in the SSN and YJD groups. Red denotes a positive association, blue denotes a negative association, and white denotes no association. *False discovery rate (FDR)‐adjusted *p* < 0.1. **FDR‐adjusted association of *p* ≤ 0.01. ***FDR‐adjusted association of *p* ≤ 0.001.

We also conducted taxonomic annotation of the bacteria expressing the genes corresponding to these KOs and found that the class Gammaproteobacteria and the family Enterobacteriaceae were the principal taxa expressing the genes related to the various different metabolites related to KOs (Figure 6A).

### 3.3 Relationships of Urinary Metabolite Concentrations With the Intestinal Microbiome

To further evaluate the relationships between gut bacteria and urinary metabolite concentrations, we calculated Spearman's correlations between the concentrations of 14 metabolites (Figure 2) and the abundances of 76 microbial taxa (33 families and 44 genera) that were present at > 0.01%. Eight families and eight genera were significantly associated with the metabolites, with *q* < 0.1. As shown in Figure 6A, these metabolites were principally associated with Fusobacteriaceae, Enterobacteriaceae, Prevotellaceae, and *Dorea*. For example, the amino acid Leu exhibited positive correlations with the genera *Dorea* and *Prevotella* and the family Prevotellaceae; and Trp and Tyr showed positive correlations with *Dorea* and Fusobacteriaceae (all *q* < 0.1). Conversely, these amino acids showed negative correlations with Enterobacteriaceae and Peptostreptococcaceae (*q* < 0.1). 2‐AB was closely negatively associated with Bacteroidaceae and *Bacteroides* (*q* < 0.001) and positively correlated with Fusobacteriaceae (*q* < 0.05), Prevotellaceae, and the genera *Prevotella* and *Dorea* (*q* < 0.01). Pyruvate was positively associated with Prevotellaceae, *Dorea*, and Fusobacteriaceae, and negatively associated with Enterobacteriaceae (*q* < 0.1). Finally, O‐phosphocholine and TMAO negatively correlated with Streptococcaceae (*Streptococcus*) and *Megamonas* (*q* < 0.1).

## 4 Discussion

Dietary nutrients affect physiological and pathological processes by affecting the microbiota and host metabolism and immune function (Zmora et al. 2019; Wu et al. 2022). Yogurt contains various nutrients, living bacteria, and many other compounds, and epidemiological studies have shown that yogurt consumption is negatively associated with the risks of obesity, diabetes, and metabolic syndrome (Buziau et al. 2019; Miller et al. 2021; Panahi et al. 2018; Watanabe et al. 2018; Cormier et al. 2016). Urine samples are commonly used for metabolomic studies because they contain a variety of metabolites that reflect the health status of individuals, as well as exogenous metabolites introduced through the diet. In addition, the composition and function of the gut microbiota influence the host's metabolic response to their diet (Sonnenburg and Bäckhed 2016). In the present study, we compared the metabolic response of healthy adults to SSN and YJD by analyzing urinary metabolites using NMR. The relationships of urinary metabolite concentrations with the abundances of intestinal microbes, analyzed using 16S rRNA amplicon sequencing, were also assessed. The metabolic and gut microbial signatures of the participants differed significantly after the consumption of the two yogurt products. There were also marked changes in the concentrations of several metabolites (MA, OPC, TMAO, 3‐HIB, fumarate, Trp, and urea) and various gut microbial functions associated with biochemical pathways (Tables S1 and S2).

Fumarate and pyruvate are key energy metabolites in several metabolic pathways, including the TCA cycle, pyruvate metabolism, glycolysis, and gluconeogenesis. These two metabolites exhibited different changes, with the urinary concentrations increasing in the SSN group and decreasing in the YJD group, such that there were significantly higher concentrations in the SSN group on d28 (*p* < 0.05, Table S1). Zheng et al. (2015) found that an accumulation of fumarate can be the result of gene mutations that cause inactivation of the enzyme fumarate hydratase in the TCA cycle. In the present study, we found that the expression of the fumarate reductase subunit D gene (frdD, K00242) was lower in both groups after yogurt consumption, which may have resulted in less conversion of fumarate. Pyruvate is the starting point of the TCA cycle and is the metabolite at the intersection of carbohydrate, lipid, and amino acid metabolic pathways. In the present study, the opposite changes in pyruvate concentration in the two groups, accompanied by two‐fold changes in lactate, acetate, and succinate concentrations in the SSN group, imply that there are differences in the effects of the SSN and YJD products on energy metabolism. A previous study performed in a mouse model showed that the concentrations of three energy metabolites (fumarate, pyruvate, and lactate) correlated with the abundances of gut bacterial taxa, such as *Gemella, Bifidobacterium*, and *Roseburia* (Cui et al. 2021). Significant associations with several gut bacterial taxa were identified in the present study, including significant alterations in the abundance of Enterobacteriaceae, which express the K01753, K01758, and K00242 genes, in the SSN group (Figures 4 and 6).

Amino acids play multiple roles in tissue structure and function, but increases in the serum concentration of amino acids are closely associated with metabolic disorders. Trp is an essential amino acid in humans that is obtained exclusively from the diet, and the free Trp concentration is determined by the balance between food intake and Trp metabolism. A previous study showed yogurt consumption increases the serum concentrations of some amino acids, including Trp and Tyr, vs. that of milk (Pimentel et al. 2018). In the present study, urinary Trp excretion was significantly lower after YJD consumption and was lower than in the SSN group on d28, but there was no clear change in the SSN group (Table S1). This may be explained by the greater use of free Trp by lactic acid‐generating bacteria during fermentation (Gummalla and Broadbent 1999), which would lead to lower concentrations of free Trp in the YJD product and lower urinary concentrations of Trp after YJD consumption, but not following SSN consumption. It is important to note that the metabolism of Trp by the gut microbiota may also contribute to the urine concentration of Trp.

Approximately 4%–6% of the Trp obtained from the diet is metabolized to indole, kynurenine, and other derivatives by gut microbes, including *Bifidobacterium*, *Lactobacillus*, *Clostridium*, *Bacteroides*, and *Peptostreptococcus* (Su et al. 2022; Roager and Licht 2018; Agus et al. 2018). These bacteria might encode enzymes in the indole/kynurenine pathway of Trp metabolism, such as amidase, kynureninase, and decarboxylase. In the present study, the expression of amidase (K01426), kynureninase (K01556), tryptophan 2,3‐dioxygenase (K00453), and indole pyruvate decarboxylase (K04103) genes, which were mainly identified in Enterobacteriaceae, Coriobacteriaceae, and Betaproteobacteria using PICRUSt2, was significantly influenced by the consumption of the SSN and YJD products (Table S2). Further analysis revealed that the urinary concentration of Trp was associated with the abundances of *Dorea*, *Bifidobacterium*, Peptostreptococcaceae, and Enterobacteriaceae (Figure 6A). The urinary concentrations of Tyr were significantly lower after the consumption of YJD than SSN, and were closely associated with the abundance of Enterobacteriaceae, with high expression of D‐amino‐acid dehydrogenase (dadA, K00285) and catalase–peroxidase (katG, K03782) (*q* < 0.1, Figure 6A).

Urea is the major end product of protein metabolism, and 80%–90% of nitrogen is eliminated in this form. Both the plasma and urinary urea concentrations represent the balance of protein or amino acid supply, absorption, and oxidation (Shao et al. 2020). In the present study, the urea concentration in the SSN group was not significantly affected by yogurt consumption, whereas in the YJD group, it was significantly higher on d28 than on d0 (*p* < 0.05), and this was accompanied by a two‐fold higher creatine concentration in the YJD group on d28 than at baseline or in the SSN group on d28, suggesting that YJD consumption affects the urea cycle. In addition, the expression of the allophanate hydrolase gene (K01457), which encodes a key enzyme in the urea metabolism replacement pathway, was significantly reduced by YJD consumption. These changes indicate differences in the effects of the yogurts on amino acid metabolism, and, in particular, arginine biosynthesis, Trp metabolism, and histidine metabolism, as well as on gut microbial function.

TMAO concentration is associated with various diseases, such as cardiovascular diseases and cardiorenal disorders (Gatarek and Kaluzna‐Czaplinska 2021). However, Hoyles found that TMAO protects cerebrovascular and cognitive function (Hoyles et al. 2021). TMAO can be produced by the gut microbiota from various dietary compounds, and its concentration is affected by diet, the intestinal bacteria, kidney function, and liver flavin monooxygenase activity. The compounds OPC, TMAO, and MA comprise a class of metabolites that are synthesized from their precursors by bacteria. OPC is an intermediate in the synthesis of phosphatidylcholine/choline, and the latter can be converted to trimethylamine (TMA) by intestinal microbes, then further metabolized to TMAO or degraded to amines, such as MA. A diet rich in TMA precursors, such as phosphatidylcholine/choline, carnitine, and betaine, can increase the formation of TMA and TMAO (Gatarek and Kaluzna‐Czaplinska 2021). Dairy products usually contain large amounts of these precursors, and TMAO is found in the urine after the consumption of dairy products (Burton et al. 2020). In the present study, the urinary TMAO concentration was increased after the consumption of both SSN and YJD, but the concentration in the SSN group at the end of the study remained lower than that in the YJD group (Table S1). Indeed, TMAO precursors are found in full‐fat dairy products (Burton et al. 2020).

Furthermore, several associations were identified between the TMAO concentration and the abundances of choline‐degrading bacteria, including bacteria from the Tenericutes phylum, the Clostridiaceae, Peptostreptococcaceae, Porphyromonadaceae, and Lachnospiraceae families, and the *Desulfovibrio, Acinetobacter*, and *Klebsiella* genera (Burton et al. 2020; Koeth Robert et al. 2013; Ji et al. 2021). The abundance of *Megamonas* and the expression of TMAO reductase (K03532, K03533, and K07811) were significantly reduced by SSN consumption, and the former was negatively associated with the TMAO concentration (*q* < 0.05) in the present study. In addition, *Faecalibacterium*, the Bifidobacteriaceae family, and its member *Bifidobacterium* were also associated with the TMAO concentration (*p* < 0.05). This suggests that an imbalance in the expression of enzymes such as choline TMA‐lyase and TMAO demethylase was present at the genus level and among four bacterial phyla (Martínez‐del et al. 2015).

MA is also derived from dietary precursors through bacterial metabolism, and its concentration can be increased by seafood and creatine supplementation (Mitchell and Zhang 2001; Poortmans et al. 2005). In the present study, the MA and O‐phosphocholine concentrations were significantly higher after the consumption of SSN. Seafood consumption was noted to be higher in the SSN group (Table 1), and we hypothesize that these higher levels of MA and O‐phosphocholine may be associated with the higher creatinine concentration in SSN (Jiang et al. 2022) and/or the higher seafood intake.

Previous studies have focused primarily on characterizing the differences in the composition of the human gut microbiota following the consumption of brown yogurt or probiotic yogurt, and there have been few comparisons of the effects of both types of yogurt. We found that SSN principally affected ammonia metabolism, whereas YJD principally affected amino acid metabolism. In contrast to the processing that is involved in the production of traditional yogurt and related products, the production of brown yogurt involves a long‐term, high‐temperature baking procedure that promotes browning before fermentation. As well as creating a brown color, this process generates differences in taste, flavor, and the constituent compounds. Therefore, the effects of brown yogurt consumption on the gut microbiota may differ from those of traditional fermented milk.

There were some limitations to the present study. Other parameters, such as renal function, diet composition, and long‐term or previous dietary habits of the participants, could have affected the urinary excretion of metabolites; therefore, these factors should be considered when evaluating the relationship between diet and the gut microbiota. In addition, we studied a relatively small number of participants and did not conduct a serum metabolite analysis. Instead, we predicted microbial metabolic function using the PICRUSt2 results, and therefore, these were theoretical, rather than based on actual expression levels. The data generated regarding transient expression differences, post‐transcriptional regulation, and actual metabolite concentrations require validation through subsequent transcriptomic, metabolomic, and/or metagenomic sequencing studies. Thus, further studies are needed to thoroughly investigate the mechanisms underlying the observed effects of the two yogurt types on metabolism.

## 5 Conclusion

The interplay between the diet and the microbiota is key in the successful implementation of personalized nutrition because the gut microbiota significantly affects responsiveness to nutritional interventions. The results of the present study demonstrate that changes in the composition and function of the gut microbiota might modulate the metabolisms of both the gut bacteria and their hosts, resulting in differing concentrations of urinary metabolites after the consumption of the SSN and YJD yogurt products for 28 days. Different metabolic responses to yogurt consumption were identified: SSN principally altered energy metabolite concentrations and TMAO metabolism, whereas YJD principally modified amino acid metabolism. These findings provide potentially useful information regarding the implementation of precision nutrition. However, the mechanisms by which the two types of yogurt affect metabolism remain incompletely understood and require further investigation.

## Author Contributions

Tie Min Jiang: conceptualization, methodology, writing – review and editing, funding acquisition. Si Ting Chen: data curation, formal analysis, visualization. Yu Chun Wang: data curation, formal analysis. Xia Qi Xiong: investigation, project administration. Jun Ying Zhao: conceptualization, methodology. Li Jun Chen: conceptualization, methodology, project administration, supervision, funding acquisition.

## Conflicts of Interest

Tie Min Jiang, Si Ting Chen, and Yu Chun Wang are employees of Guilin University of Technology. Jun Ying Zhao and Li Jun Chen are employees of Beijing Sanyuan Foods Co. Ltd. Xia Qi Xiong is an employee of Liuzhou Sanyuan Foods Co. Ltd.

## Supporting information


**Table S1:** Differences in urinary metabolite concentrations after consumption of different yogurts.

**Table S2:** Enrichment KEGG pathway of different KOs and enrichment KOs related to different metabolites after yogurt intaking.

## Data Availability

The data that support the findings of this study are available on request from the corresponding author. The data are not publicly available due to privacy or ethical restrictions.
